# New Drugs. Appliances, and Things Medical

**Published:** 1892-04-02

**Authors:** 


### NEW DRUGS, APPLIANCES, AND THINGS MEDICAL.

[All preparations' appliances, novelties, etc., of which a notice is desired, should be sent for The Editor, to care of The Manager, 140, Strand, London, W.C.]

### IMPROVEMENT IN SYPHONS.

Messrs. Barnett and Foster, Eagle Wharf Road, New North Road, have recently patented a very great improvement in syphons. Ordinarily, when the tip of a syphon is opened, the liquid comes out with such violence that it overruns the glass and effervesces so violently that a large portion of the gas escapes, and the liquid that remains is correspondingly flat. This invention obviates these faults. It consists of a perforated elastic washer, moving centrally downwards in a conoidal chamber at the head of the syphon tube. It will be seen that charging the syphon is quite easy, as the washer expands downwards with the pressure, and the central aperture enlarges with the pressure, so that practically there is no opposing force to the incoming charged fluid. On opening the tap of one of these syphons, the pressure forces the washer against its collar, and the fluid thus escapes through a narrow elastic regulating opening into the larger tube above. There is thus always a regular flow from the tap instead of the impetuous overflow of the ordinary form. We are decidedly of opinion that this check valve will prove a great success.

